# Prognostic role of tumor-infiltrating CD57-positive lymphocytes in solid tumors: a meta-analysis

**DOI:** 10.18632/oncotarget.23621

**Published:** 2017-12-22

**Authors:** Guoming Hu, Shimin Wang

**Affiliations:** ^1^ Department of General Surgery, Breast and Thyroid Surgery, Shaoxing People's Hospital, Shaoxing Hospital of Zhejiang University, 312000, Zhejiang, China; ^2^ Department of Nephrology, Shaoxing People's Hospital, Shaoxing Hospital of Zhejiang University, 312000, Zhejiang, China

**Keywords:** tumor-infiltrating CD57^+^ lymphocytes, favorable outcome, solid tumor, meta-analysis

## Abstract

The prognostic role of tumor-infiltrating CD57-positive lymphocytes (CD57^+^ lymphocytes) in human solid tumors remains controversial. Herein, we conducted a meta-analysis including 26 published studies with 7656 patients identified from PubMed and EBSCO to assess the prognostic impact of tumor-infiltrating CD57^+^ lymphocytes in human solid tumors. We found that CD57^+^ lymphocyte infiltration significantly improved overall survival (OS) including 1 – year, 3 – year and 5 – year survival, and disease – free survival (DFS) in all types of solid tumors. In stratified analyses, CD57^+^ lymphocyte infiltration was significantly associated with better OS in hepatocellular, esophageal, head and neck carcinoma, non-small cell lung cancer, 5 – year survival in colorectal cancer, and 3 – year and 5 – year survival in gastric cancer, but not with 1 – year survival in gastric cancer, or 1 – year or 3 – year survival in colorectal cancer. In addition, high density of intratumoral CD57^+^ lymphocytes was significantly inversely correlated with lymph node metastasis and TNM stage of solid tumor. In conclusion, CD57^+^ lymphocyte infiltration leads to a favorable clinical outcome in solid tumors, implicating that it is a useful biomarker for prognosis and adoptive immunotherapy based on these cells may be a promising choice for treatment.

## INTRODUCTION

Tumor microenvironment (TME) has proven to be closely related to the development and progression of cancer through diverse mechanisms including promoting immune suppression and stimulating angiogenesis [1]. Tumor-infiltrating immune cells (TICs) are considered to be the important components of TME [2]. Previous studies have revealed that TICs provided prognostic values in several types of solid tumors [3]. However, it is important to distinguish among a variety of immune cells as they may play differential roles in the TME. As the important component of adaptive immunity, the functions of CD57-positive lymphocytes in TME have drawn much attention in recent decades. Furthermore, these lymphocytes have been demonstrated to play significant roles in a number of human cancers.

CD57, also called human natural killer-1 (HNK-1) or LEU-7, has been shown to be present on CD4^+^ T, especially on CD8^+^ T and natural killer (NK) cells at the late stages of differentiation. It is generally applied to recognize the terminally differentiated cells with lower proliferative capacity and altered functional activities. CD57-positive lymphocytes (CD57^+^ lymphocytes) are often increased in solid tumors. Recent studies have associated tumor-infiltrating CD57^+^ lymphocytes and cancer prognosis, but their results were controversial [4]. Thus, the value of these cells as a biomarker for prognosis and related immunotherapy need further assessment.

Herein, we performed this meta-analysis to quantitatively explore the correlation between high density of CD57^+^ lymphocytes and clinical outcomes such as overall survival (OS) and disease–free survival (DFS), thereby provided more evidence on the clinical value of CD57^+^ lymphocytes for prognostic prediction for solid tumors.

## RESULTS

### Search results

Total 9664 Literatures were searched and the results were displayed in Supplementary Figure 1. 26 studies containing 7656 patients with solid tumor were identified for the assessment of tumor-infiltrating CD57^+^ lymphocytes [5–30]. All included studies were assessed using Newcastle–Ottawa Scale (NOS), and met the inclusion criteria. Characteristics of these studies for OS, DFS and clinicopathological features including TNM stage etal have been exhibited in Table 1 and Supplementary Table 1 respectively.

**Table 1:** Main characteristics of the included studies

Study	Year	Tumor type	No. of Patients	Male/Female	median age (range) (year)	CD57^+^ lymphocytes: High/Low	Tumor stage	median follow-up date (months)	Survival	Quality Score (NOS)
Chen, Y.F et al. [5]	2016	Colorectal cancer	300	158/142	< 60: 48%; ≥ 60: 52%	NR	I–IV	62.9 ± 29.3	OS, DFS	6
Chaput, N. et al. [6]	2013	Colorectal cancer	196	108/88	(27, 86)	56/100	II–III	≥ 24	OS, DFS	7
Liska, V. et al. [7]	2012	Colorectal cancer	150	93/57	NR	34/11	NR	NR	OS	6
Sconocchia, G. et al. [8]	2011	Colorectal cancer	1400	NR	71 (30, 96)	189/1070	NR	NR	OS	6
Menon, A. G. et al. [9]	2004	Colorectal cancer	93	56/37	< 50: 13%; ≥ 50: 87%	30/62	II–III	73.2 (1.2, 223.2)	DFS	7
Coca, S. et al. [10]	1997	Colorectal cancer	186	110/76	63.4 (29, 84)	25/132	I–III	≥ 60	OS, DFS	6
Gao, Q. et al. [11]	2012	Hepatocellular carcinoma	206	186/20	≤ 49: 50.5%; > 49: 49.5%	NR	I–III	48.1 (3.4, 111.9)	OS, DFS	8
Zhao, J. J. et al. [12]	2014	Hepatocellular carcinoma	163	131/32	< 50: 57%; ≥ 50: 43%	82/81	NR	NR	OS	7
Liu, K. et al. [13]	2015	Gastric cancer	166	41/125	< 60: 60.8%; ≥ 60: 39.2%	83/83	I–IV	65.88	OS	8
Ishigami, S. et al. [14]	2000	Gastric cancer	169	121/48	63.8 (30, 87)	53/116	NR	63	OS	7
Ishigami, Sumiya. et al. [15]	2000	Gastric cancer	146	108/38	63.8 (30, 87)	39/107	I–IV	87	OS	7
Lv, L. et al. [16]	2011	Esophageal carcinoma	181	141/40	< 60: 58%; ≥ 60: 42%	91/90	I–IV	NR	OS	8
Hsia, J. Y. et al. [17]	2005	Esophageal carcinoma	38	38/0	< 60: 47.4%; ≥ 60: 52.6%	14/24	I–IV	NR	OS	6
Fang, J. et al. [18]	2017	Head and neck carcinoma	78	57/21	60 (24, 82)	34/44	I–IV	48 (29, 93)	OS	7
Taghavi, N. et al. [19]	2016	Head and neck carcinoma	57	27/30	62.89 (34, 91)	26/31	NR	29 (10, 85)	OS	7
Fraga, C. A. et al. [20]	2012	Head and neck carcinoma	70	61/9	54.9 (36, 88)	35/35	NR	NR	OS	6
Karpathiou, G. et al. [21]	2017	Head and neck carcinoma	152	128/24	58.5 (40, 88)	71/81	I–IV	24 (3, 84)	OS	7
Hernandez-Prieto, S. et al. [22]	2015	Non-small cell lung cancer	84	72/12	66.5 ±10.2	29/55	I–II	45.97	DFS	6
Villegas, F. R. et al. [23]	2002	Non-small cell lung cancer	50	49/1	(50, 81)	21/29	I–IIIA	(6.2, 149.3)	OS	6
Takanami, I. et al. [24]	2001	Non-small cell lung cancer	150	83/67	61 (30, 81)	53/97	I–IIIA	(60, 120)	OS	8
Donskov, F. et al. [25]	2006	Renal Cell Carcinoma	120	85/35	57 (19, 74)	34/51	IV	7 (32, 73)	OS	7
Li, K. et al. [26]	2009	Ovarian Cancer	82	0/82	(26, 80)	33/49	I–IV	(1, 154)	OS	6
Ohnishi, K. et al. [27]	2016	Endometrial Carcinoma	79	0/79	59 (30, 74)	37/38	I–IV	NR	OS	7
Hansen, B. D. et al. [28]	2006	Melanoma	27	16/11	49 (31, 65)	13/12	IV	8.9 (1, 35)	OS	6
Blaker, Y. N. et al. [29]	2016	Lymphoma	52	NR	NR	NR	NR	120 (15.6, 40.8)	OS	6
Wangerin, H. et al. [30]	2014	Prostate cancer	3261	3261/0	62	NR	NR	34 (1, 144)	OS	6

### Meta-analyses

#### OS

**Figure 1:**
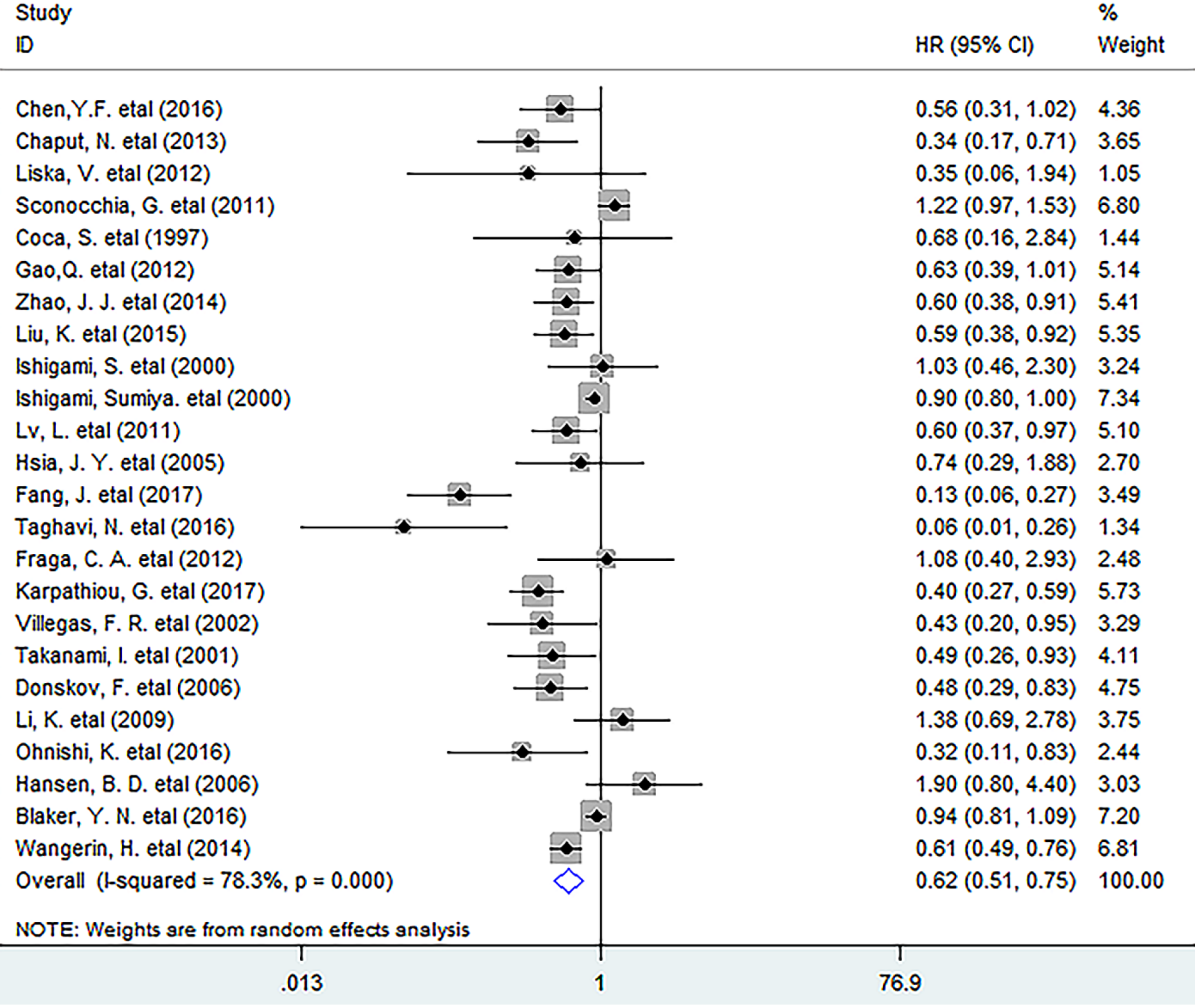
Forest plots describing HR of the association between CD57^+^ lymphocyte infiltration and OS in solid tumors

In this meta-analysis, the pooled results indicated that increased density of intratumoral CD57^+^ lymphocytes was significantly associated with better OS (HR = 0.62, 95% CI 0.51 to 0.75, *P* = 0.000) in solid tumors (Figure 1). More specifically, CD57^+^ lymphocyte infiltration significantly improved 1 – year (OR = 1.73, 95% CI 1.08 to 2.77, *P* = 0.022) and 3 – year (OR = 3.09, 95% CI 2.03 to 4.70, *P* = 0.000) as well as 5 – year (OR = 3.48, 95% CI 2.24 to 5.42, *P* = 0.000) survival rate in patients. (Supplementary Figure 2).

**Figure 2:**
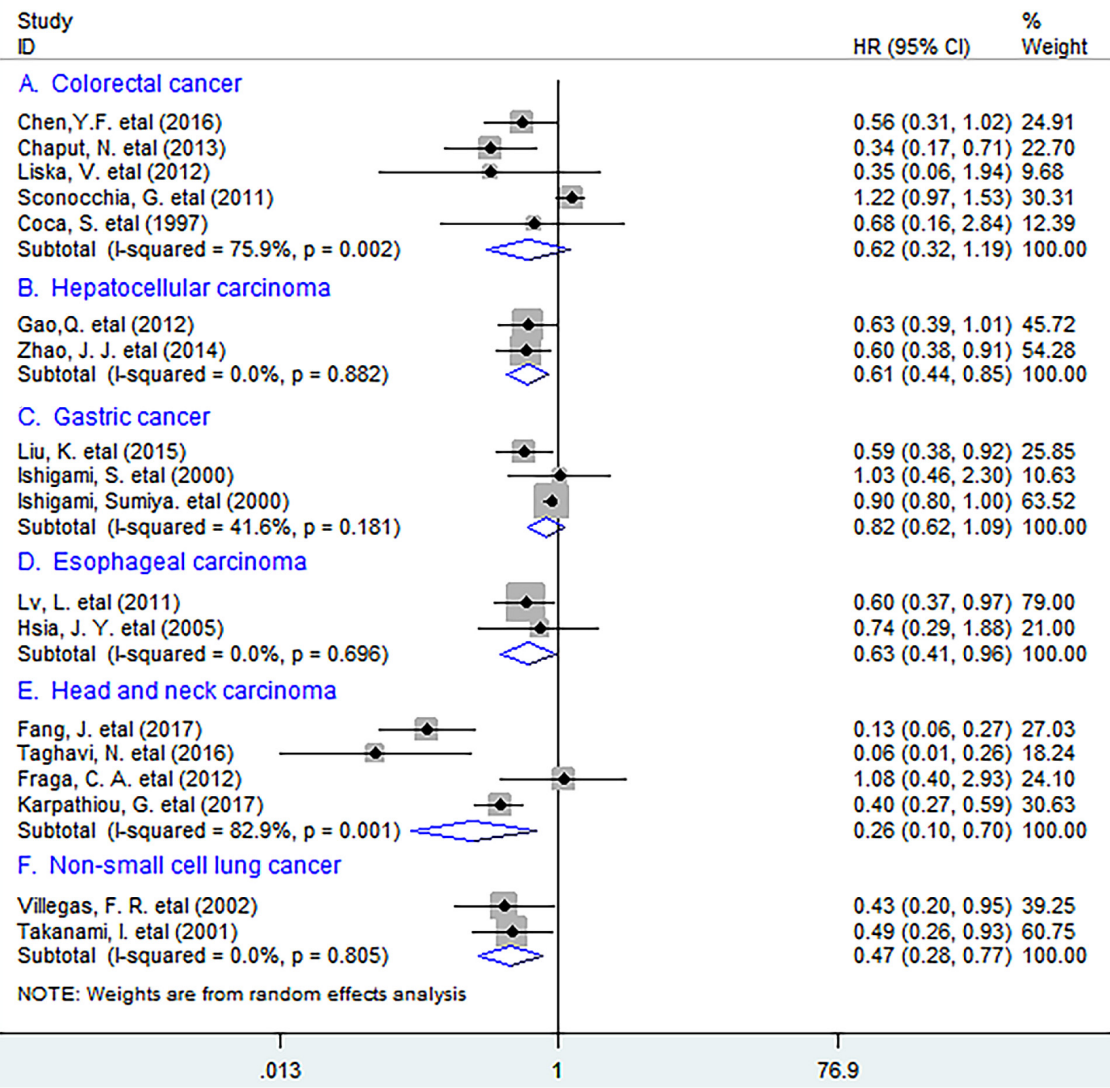
Stratified analyses describing HRs of the association between CD57^+^ lymphocyte infiltration and OS

In subgroup analyses according to the types of cancer, the results indicated that CD57^+^ lymphocytes infiltrating into tumor significantly improved OS in hepatocellular cancer (HCC) (HR = 0.61, 95% CI 0.44 to 0.85, *P* = 0.003) and esophageal carcinoma (EC) (HR = 0.63, 95% CI 0.41 to 0.96, *P* = 0.033), with no heterogeneity being found (*I^2^ = 0.0%, P = 0.882; I^2^ = 0.0%, P = 0.696 respectively*) (Figure 2). Similar results were observed between tumor-infiltrating CD57^+^ lymphocytes and OS in head and neck carcinoma (HNC) (HR = 0.26, 95% CI 0.10 to 0.70, *P* = 0.007) and non-small cell lung cancer (NSCLC) (HR = 0.47, 95% CI 0.28 to 0.77, *P* = 0.003). However, no significant correlation between CD57^+^ lymphocyte infiltration and OS in colorectal cancer (CRC) (HR = 0.62, 95% CI 0.32 to 1.19, *P* = 0.152) or gastric cancer (GC) (HR = 0.82, 95% CI 0.62 to 1.09, *P* = 0.167) was found.

**Figure 3:**
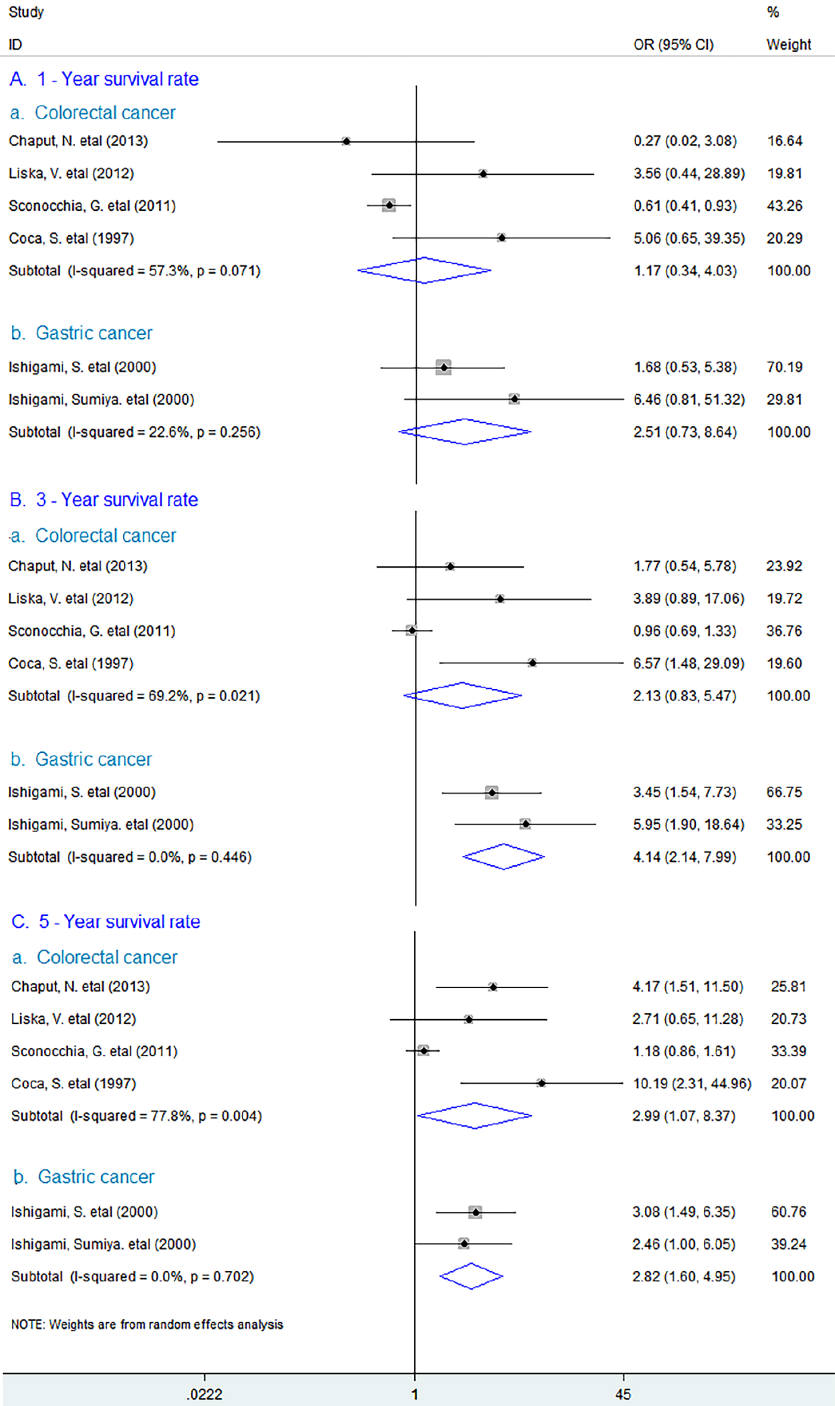
Forest plots describing ORs of the association between CD57^+^ lymphocyte infiltration and OS at 1-year, 3-year and 5-year in colorectal and gastric cancer

In addition, the study showed that increased density of CD57^+^ lymphocytes was associated with better 5 – year (OR = 2.99, 95% CI 1.07 to 8.37, *P* = 0.037), but not with 1 – year (OR = 1.17, 95% CI 0.34 to 4.03, *P* = 0.809) or 3 – year survival rate (OR = 2.13, 95% CI 0.83 to 5.47, *P* = 0.114) survival rate in CRC. Whereas in GC, CD57^+^ lymphocyte infiltration could significantly improve 3 – year (OR = 4.14, 95% CI 2.14 to 7.99, *P* = 0.000) and 5 – year (OR = 2.82, 95% CI 1.60 to 4.95, *P* = 0.000) except 1 – year survival rate (OR = 2.51, 95% CI 0.73 to 8.64, *P* = 0.144). (Figure 3)

#### DFS

**Figure 4:**
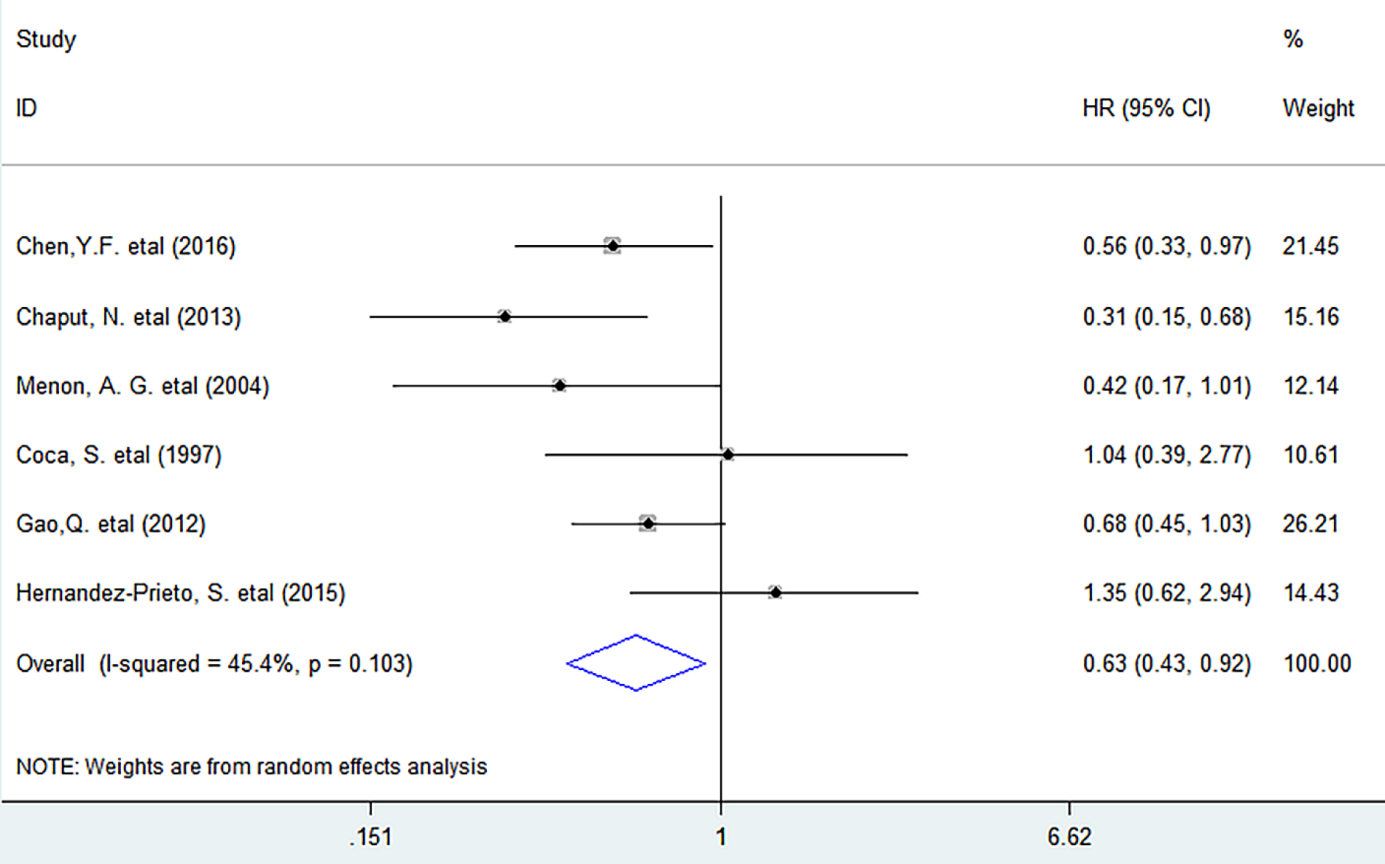
Forest plots describing HR of the association between CD57^+^ lymphocyte infiltration and DFS in solid tumors

Meta-analysis showed that CD57^+^ lymphocyte infiltration significantly improved DFS (HR = 0.63, 95% CI 0.43 to 0.92, *P* = 0.016) in solid tumors, with no significant heterogeneity existing among studies (*I^2^ = 45.4%, P = 0.103*). (Figure 4)

We next investigated whether tumor-infiltrating CD57^+^ lymphocytes correlated with clinicopathological features including lymph node metastasis etal of tumor. We found that CD57^+^ lymphocyte infiltration was significantly inversely associated with lymph node metastasis (OR = 0.36, 95% CI 0.26 to 0.50, *P* = 0.000) and TNM stage (OR = 2.14, 95% CI 1.11 to 4.11, *P* = 0.022), but not with tumor differentiation (OR = 0.78, 95% CI 0.51 to 1.17, *P* = 0.229), or vascular invasion (OR = 0.58, 95% CI 0.11 to 3.09, *P* = 0.525) of patients. (Supplementary Figure 3)

### Sensitivity analysis

Sensitivity analyses indicated that none of the studies included in the meta-analysis could significantly affect the overall HR for OS or DFS. (data not shown)

### Publication bias

Funnel plot and Egger's test showed that there was no publication bias between CD57^+^ lymphocyte infiltration and OS (*P* = 0.984) or DFS (*P* = 0.492) in patients with solid tumor.

## DISCUSSION

Lymphocytes play a vital role in protecting the host from pathogenic microorganisms especially viruses and cancer. In the last decades, although many studies have associated tumor-infiltrating CD57^+^ lymphocytes and prognosis of solid tumors, their results were not consistent even controversial. In the present meta-analysis, we found that CD57-positive terminally differentiated lymphocyte infiltration had a positive prognostic effect associated with survival in solid tumors especially in HCC, EC, HNC, NSCLC, CRC and GC. In addition, increased density of CD57^+^ lymphocytes inversely correlated with lymph node metastasis and TNM stage of solid tumor. These findings suggest that CD57^+^ lymphocytes are one of the important players in establishment of antitumor immunity in human solid tumors that retarding tumor progression.

The close association between increased CD57^+^ lymphocytes and improved survival of patients identified in this study may relate to the following reasons: first, CD57 expression in lymphocytes such as CD8^+^ T and NK cells correlates with increased expression of cytolytic enzymes such as granzyme A, granzyme B and perforin, suggesting that CD57^+^ lymphocytes possess the ability to cytolysis target cells including tumor cells [31]. Second, CD4^+^ T, CD8^+^ T and NK cells expressing CD57 antigen can produce more IFN-γ to inhibit the growth of tumor when stimulated [32]. In addition, CD57^+^ NK cells, known as one of the important components in adaptive immunity, can up-regulate MHC Class I and MHC Class II molecule on tumor cells through IFN-γ secretion to allow cytotoxic T – lymphocytes to recognize tumor – specific antigens thereby exerting antitumor effect [33]. More importantly, CD57^+^ NK cells can enhance the responses of T lymphocytes through the interactions between NK and dendritic cells (DC) that induce maturation and activation of DCs, [34]. with these cell – derived IFN-γ inducing T – cell polarization [35]. Thus, it is reasonable to speculate that the CD57^+^ lymphocytes are able to potentiate the antitumor immune responses in the TME and fight against tumor growth and spread therefore improving survival.

However, some limitations exist in this study. Morphometric analyses for CD57^+^ lymphocytes used in included studies were not consistent. In addition, we were unable to gain the pooled results for some cancers such as renal cell carcinoma and ovarian cancer etal due to the lack of enough data.

In conclusion, CD57^+^ lymphocyte infiltration leads to a favorable clinical outcome of patients with solid tumor, implicating that it is a useful biomarker for prognosis and adoptive immunotherapy based on these cells may be a promising choice for treatment.

## MATERIALS AND METHODS

### Search strategy

We searched PubMed and EBSCO for studies assessing the density of CD57^+^ lymphocytes in tumor tissue and survival in patients with solid tumor from 1996 to June 30th 2017. The searching keywords were (CD57 [Title/Abstract] OR HNK-1 [Title/Abstract] OR LEU-7 [Title/Abstract]) AND (neoplasms [Title/Abstract] OR tumor [Title/Abstract] OR cancer [Title/Abstract] OR carcinoma [Title/Abstract]) AND (prognosis [Title/Abstract] OR survival [Title/Abstract]). A total of 2900 and 6764 entries were identified in PubMed and EBSCO respectively.

### Inclusion and exclusion criteria

Inclusion criteria of the meta-analysis were: studies included must have (1) been published as original articles; (2) evaluated human subjects; (3) CD57^+^ lymphocytes in tumor specimens were measured by immunohistochemistry (IHC); (4) provided hazard ratios (HRs) or Kaplan – Meier curves of high and low CD57^+^ lymphocyte density with overall survival (OS), and/or disease – free survival (DFS); (5) published in English.

We excluded studies that were not published as full texts, including commentary, case report, conference abstracts and letters to editors, studies that not report sufficient data to estimate hazard ratios (HRs); studies that detected lymphocytes not with marker ‘CD57’, ‘HNK-1’, or ‘LEU-7’, or detected CD57^+^ lymphocytes in metastases or not in tumor tissues.

### Endpoints

OS and DFS were the endpoints used in this meta-analysis. OS was recorded as the primary endpoint, and the second endpoint was DFS. Cut-offs of CD57^+^ lymphocyte density defined by individual studies classified cancer patients into high- and low- groups.

### Data extraction

Two authors (GM.H. and SM.W.) independently reviewed and extracted data using predefined data abstraction forms from each eligible study. Extracted information included first author's name, publication year, country, number of patients, median age, gender, Tumor, Lymph Node, Metastasis (TNM) stage, tumor differentiation, time of follow-up, technique used to quantify CD57^+^ lymphocytes, and cut-off value to determine high density of these cells. OS, DFS and clinicopathological data were extracted from the text, tables, or Kaplan – Meier curves for both high and low CD57^+^ lymphocyte density groups.

### Quality assessment

The studies included in the meta-analysis were cohort studies. The quality of each included study was assessed using Newcastle–Ottawa Scale (NOS) by two independent authors [36]. The studies with 6 scores or more were classified as high quality studies. A consensus NOS score for each item was achieved.

### Statistical analysis

Extracted data were combined into a meta-analysis using STATA 12.0 analysis software (Stata Corporation, College Station, TX, USA). Statistical heterogeneity was assessed using the chi-squared based *Q*-test or the *I^2^* method [37]. Data were combined according to the random-effect model in the presence of heterogeneity [38], otherwise, the fixed-effect model was performed [39]. Sensitivity analysis was employed to assess the influence of each study on the pooled result. Begg's funnel plot and Egger's test [40]. were calculated to investigate potential publication bias. All *P* values were two-sided and less than 0.05 are considered statistically significant.

## SUPPLEMENTARY MATERIALS FIGURES AND TABLE



## Abbreviations

OS: overall survival
DFS: disease-free survival
HR: hazard ratio
OR: odds ratios
Cl: confidence interval
TNM: Tumor
HCC: hepatocellular carcinoma
EC: esophageal carcinoma
HNC: head and neck carcinoma
NSCLC: non-small cell lung cancer
CRC: colorectal cancer
GC: gastric cancer
IHC: immunohistochemistry
NR: not reported.
