# Supplementary material for: Prognostic role of tumor-infiltrating CD57-positive lymphocytes in solid tumors: a meta-analysis
Source: Oncotarget. 2017 Dec 22;9(8):8111–9. doi: 10.18632/oncotarget.23621 (PMC5814286; doi:10.18632/oncotarget.23621)
Supplement: Supplementary file 1 [file oncotarget-09-8111-s001.pdf]

Prognostic role of tumor-infiltrating CD57-positive lymphocytes in solid tumors: a meta-analysis

SUPPLEMENTARY MATERIALS

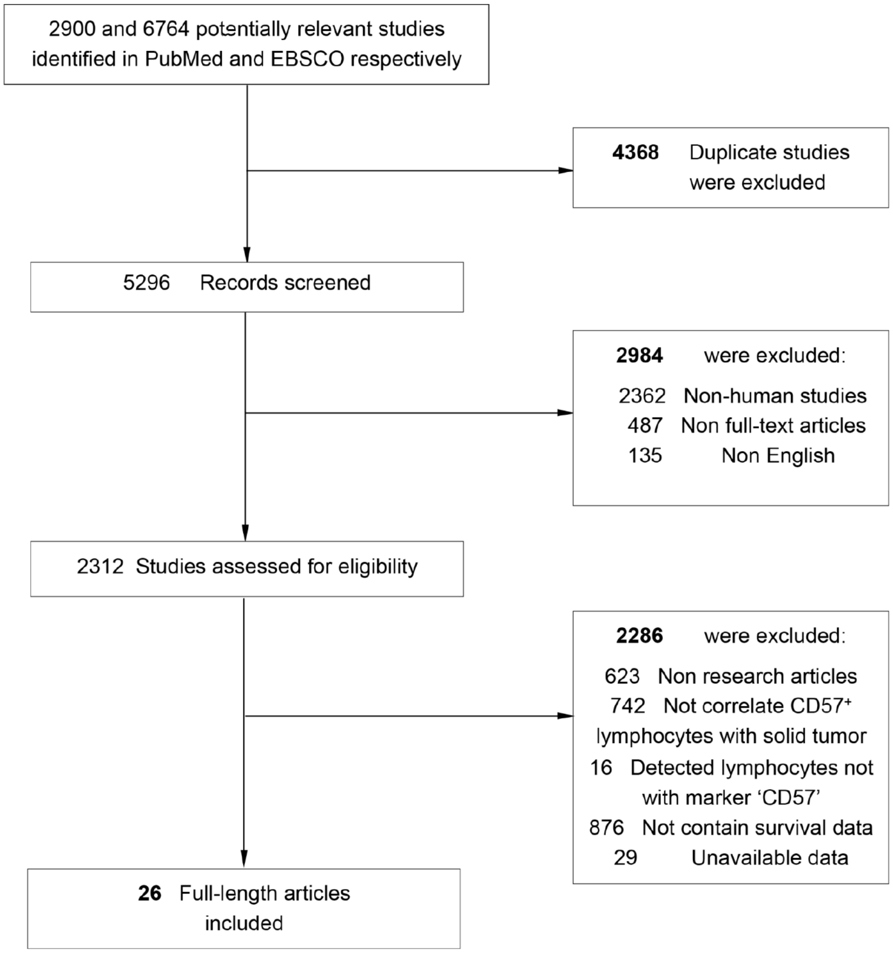

Supplementary Figure 1: Flow chart diagram of study selection.

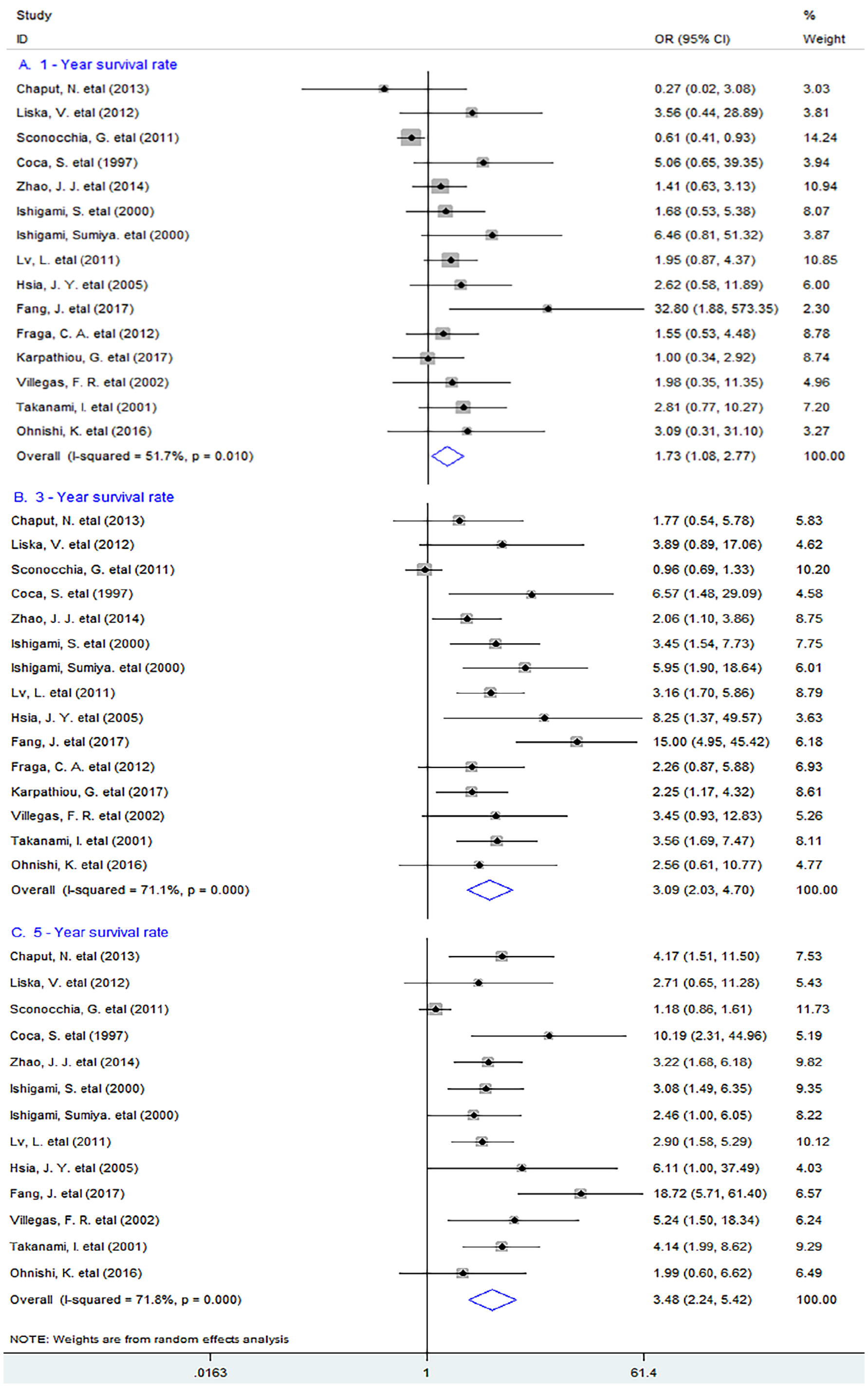

Supplementary Figure 2: Forest plots describing ORs of the association between CD57^{+} lymphocyte infiltration and OS at 1-year, 3-year and 5-year of patients with solid tumor.

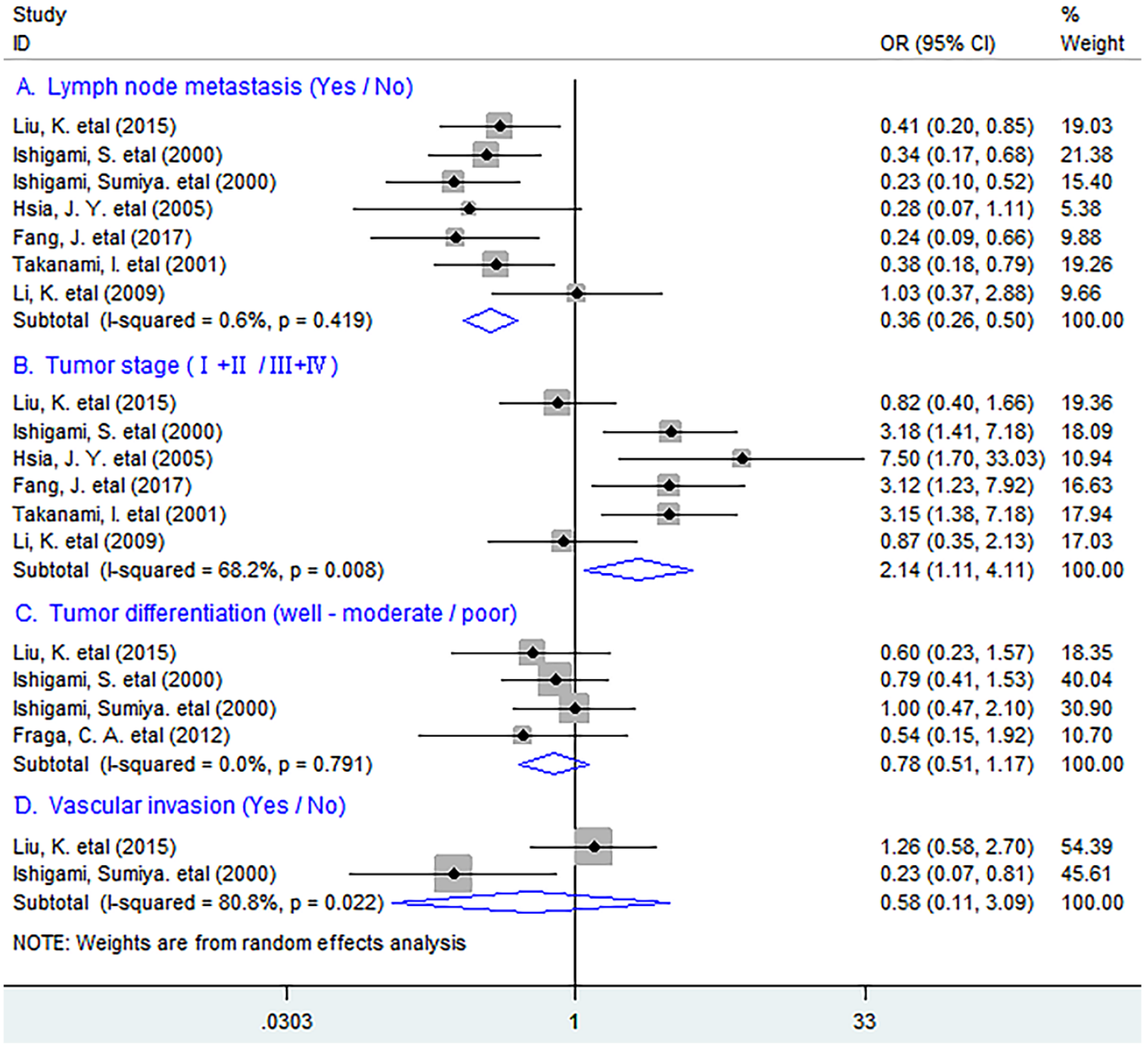

Supplementary Figure 3: Forest plots indicating ORs of the association between CD57[+] lymphocyte infiltration and clinicopathological features of solid tumor.

Supplementary Table 1: Characteristics of the included studies for OR analysis of clinicopathological features

Study	Year	Tumor type	No. of Patients	CD57$^+$ lymphocytes: High/Low	Lymph node metastasis (Yes / No)	Tumor stage	I + II / III + IV	Vascular invasion (Yes / No)	Tumor Differentiation (Well-moderate/ poor)
Liu, K. et al. [13]	2015	Gastric cancer	166	83/83	H: (54/29); L: (64/19)	I–IV	H: (39/44) L: (25/58)	H: (18/65); L: (15/68)	H: (10/30); L: (14/25)
Ishigami, S. et al. [14]	2000	Gastric cancer	169	53/116	H: (16/37); L: (65/51)	NR	NR	NR	H: (29/24); L: (70/46)
Ishigami, Sumiya. et al. [15]	2000	Gastric cancer	146	39/107	H: (10/29); L: (64/43)	I–IV	H: (29/10) L: (51/56)	H: (3/36); L: (28/78)	H: (23/16); L: (62/43)
Hsia, J. Y. et al. [17]	2005	Esophageal carcinoma	38	14/24	H: (5/9); L: (16/8)	I–IV	H: (10/4) L: (6/18)	NR	NR
Fang, J. et al. [18]	2017	Head and neck carcinoma	78	34/44	H: (7/27); L: (23/21)	I–IV	H: (21/13) L: (15/29)	NR	NR
Fraga, C. A. et al. [20]	2012	Head and neck carcinoma	70	35/35	NR	NR	NR	NR	H: (7/10); L: (13/10)
Takanami, I. et al. [24]	2001	Non-small cell lung cancer	150	53/97	H: (14/39); L: (47/50)	I–IIIA	H: (44/9) L: (59/38)	NR	NR
Li, K. et al. [26]	2009	Ovarian Cancer	82	33/49	H: (8/24); L: (12/37)	I– IV	H: (13/20) L: (21/28)	NR	NR

H: high; L: low; NR: not reported.
